# Assessing Health-Related Quality of Life in Non-Directed Versus Directed Kidney Donors: Implications for the Promotion of Non-Directed Donation

**DOI:** 10.3389/ti.2024.12417

**Published:** 2024-01-12

**Authors:** Assaf Vital, Maya Siman-Tov, Gadi Shlomai, Yana Davidov, Keren Cohen-Hagai, Moshe Shashar, Enosh Askenasy, Ronen Ghinea, Eytan Mor, Tammy Hod

**Affiliations:** ^1^ Arrow Program for Medical Research Education, Sheba Medical Center, Tel-Hashomer, Israel; ^2^ Adelson School of Medicine, Ariel University, Ariel, Israel; ^3^ Department of Emergency and Disaster Management, School of Public Health, Faculty of Medicine, Tel-Aviv University, Tel Aviv, Israel; ^4^ Faculty of Medicine, Tel-Aviv University, Tel Aviv, Israel; ^5^ Department of Internal Medicine D and Hypertension Unit, Division of Endocrinology, Diabetes and Metabolism, Sheba Medical Center, Tel-Hashomer, Israel; ^6^ Liver Disease Center, Sheba Medical Center, Tel Hashomer, Israel; ^7^ Department of Nephrology and Hypertension, Meir Medical Center, Kfar Saba, Israel; ^8^ Department of Nephrology and Hypertension, Laniado Hospital, Netanya, Israel; ^9^ Renal Transplant Center, Sheba Medical Center, Tel-Hashomer, Israel

**Keywords:** non-directed kidney donors, living kidney donors, directed kidney donors, quality of life, length of stay

## Abstract

Living kidney donation has increased significantly, but little is known about the post-donation health-related quality of life (HRQoL) of non-directed donors (NDs) vs. directed donors (DDs). We thus examined the outcomes of 112 living kidney donors (82 NDs, 30 DDs). For the primary outcomes—namely, the mean physical component summary (PCS) and mental component summary (MCS) scores of the 12-item Short Form Survey (SF-12) questionnaire—scores were significantly higher for the NDs vs. the DDs (PCS: +2.69, MCS: +4.43). For secondary outcomes, NDs had shorter hospital stays (3.4 vs. 4.4 days), returned to physical activity earlier (45 vs. 60 days), exercised more before and after donation, and continued physical activity post-donation. Regression analyses revealed that donor type and white blood cell count were predictive of the PCS-12 score, and donor type was predictive of the MCS-12 score. Non-directed donation was predictive of a shorter hospital stay (by 0.78 days, *p* < 0.001) and the odds of having PCS-12 and MCS-12 scores above 50 were almost 10 and 16 times higher for NDs, respectively (*p* < 0.05). These findings indicate the safety and potential benefits of promoting non-directed donation. However, careful selection processes must be maintained to prevent harm and exploitation.

## Graphical Abstract

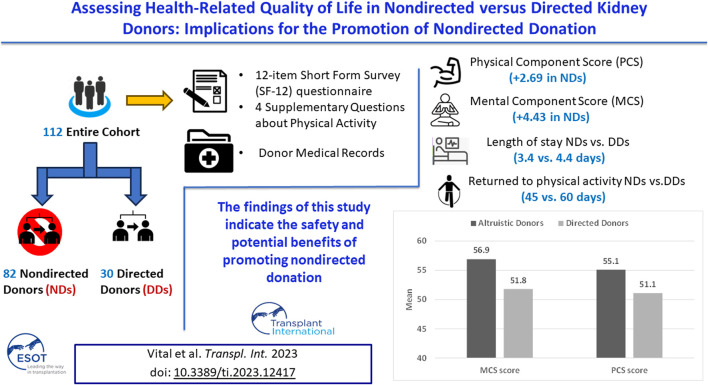

## Introduction

### Prelude

I [Assaf Vital] am a 28 years-old medical student, currently in my third year of studies at Ariel University in Israel. At the age of 16, I was diagnosed with stage 4 chronic kidney disease, which has remained stable to this day. My nephrologist advised me that at some point in the future, I would likely need a kidney transplant, and I should start looking for a donor. The thought of asking someone for such a major gift was daunting, and I felt I needed to understand more about what it would entail before doing so.

Under the guidance of Dr. Hod, I undertook research to explore the implications of kidney donation on the lives of donors, both in terms of their physical health and their mental wellbeing. Through my investigations, I hope to provide physicians and patients with a clearer understanding of what donation involves and what the potential consequences might be. As part of my research, I spoke with several individuals who had donated a kidney to a loved one or to a stranger. Their insights and experiences gave me a deeper appreciation of the sacrifices involved in kidney donation, as well as the extraordinary generosity and resilience of the donors themselves. For instance, I remember speaking with one donor who apologized for being breathless on the phone since she had just finished a half-marathon with a group of other kidney donors. Another donor shared with me that recovering from laser eye surgery had been more difficult than recovering from kidney donation.

Ultimately, my research helped me to feel more informed and empowered in facing my own kidney transplant journey. While I have not yet found a donor, “I am heartened by the knowledge that there are many compassionate and courageous people out there who are willing to give the gift of life to others.”

### Background

The rate of living kidney donation has increased significantly over the years, accounting for a global increase to 38% of all kidney transplants in 2021 [1]. This welcome trend is helping to bridge the gap between the shortage of deceased donor organs and the growing number of transplant candidates on waiting lists. In addition, there are clear advantages of living over deceased kidney donation, including minimization of the recipient’s waiting time and shorter cold and warm ischemic times, with consequent improved graft quality and transplant outcomes. An additional advantage is that the surgery is elective, enabling optimization of the recipient’s health before the transplant [2–6].

Living kidney donation may be directed or non-directed. Directed kidney donation is donation to a recipient with whom the donor has a genetic and/or emotional relationship pre-transplant, while non-directed kidney donation is donation to a recipient with whom the donor has no previous acquaintance. It is notably more straightforward for medical professionals and policymakers to endorse directed kidney donation, where a family member, close friend, or anyone with an emotional connection to the recipient donates a kidney out of a sense of obligation or personal will. However, non-directed kidney donation presents a distinct challenge.

The number of non-directed donors has increased sharply in recent years [7], contributing significantly to the feasibility of kidney paired or pooled exchange programs and facilitating transplants for high immunological risk recipients [8, 9]. Yet, clinicians express skepticism about motivations for non-directed donation and concerns about long-term physical and psychological outcomes for non-directed donors and hence hesitate to actively promote it [10–12]. Thus, non-directed kidney donation remains uncommon, being limited to a minority of European countries due to legal constraints and moral objections and accounting for only 10% and 3% of all living donations in the United Kingdom and the United States, respectively [13].

In Israel, a non-profit organization, known as Matnat Chaim (meaning the Gift of Life), has emerged as a major force encouraging living—mainly non-directed—kidney donation. The organization has facilitated 1,398 live kidney donations since its founding in February 2009 (up to the end of February 2023), thereby contributing to a steady increase in the number of living kidney donations per year in Israel from 71 in 2010 to 319 in 2022. These 319 living donations comprised 68.75% of the total of 464 kidney transplants in Israel in 2022, with non-directed donors contributing 58.3% (186/319) of the kidneys.

To shed light on the dilemma of whether living kidney donation, specifically non-directed kidney donation, should be encouraged, this study aimed to evaluate the health-related quality of life (HRQol) of living donors after donation. Specifically, we compared the HRQol of directed vs. non-directed donors, alongside examining differences between the two groups in hospital length of stay (LOS), time to return to normal activity, and time to physical activity post donation.

## Materials and Methods

### Study Population

The study is of a cross sectional design. All 179 individuals who underwent laparoscopic kidney donation at the Sheba Medical Center between the end of June 2019 and the beginning of October 2022 were eligible to participate in the study. Three donors were excluded, one due to first year recipient graft loss and two due to deaths of the recipients in the first year after transplant. A total of 176 donors—130 non-directed and 46 directed—were contacted via phone and asked to participate in the study. Donors who consented were required to confirm receipt of our questionnaire—based on SF-12 plus four Supplementary Questions—via WhatsApp or email through a Google form. Participants were provided with a designated phone number for assistance with questionnaire completion or for any queries.

**FIGURE 1:**
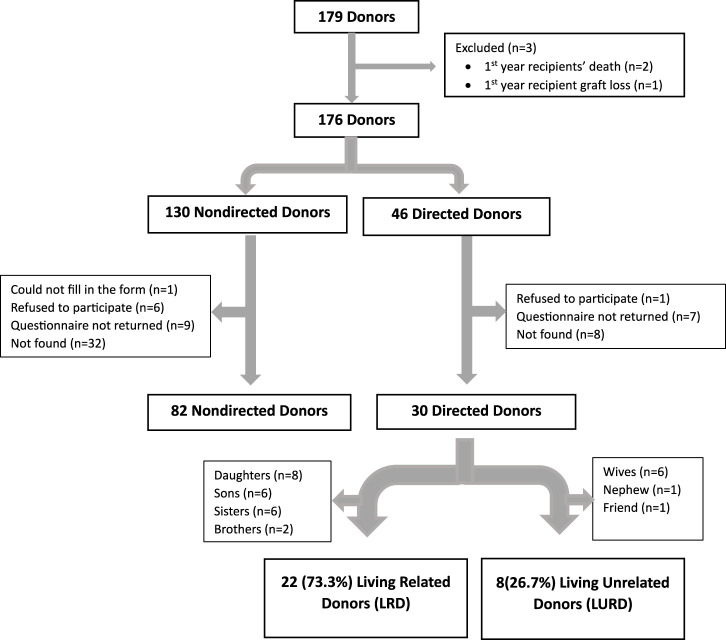
Consort diagram.

Eighty-two (63.1%) of the 130 non-directed donors and 30 (65.2%) of the 46 directed donors returned the completed study questionnaires and comprised our final study cohort (Figure 1). A comparison of age, sex, and year of donation between study participants and non-participants showed no significant differences. Similarly, there were no significant differences in the participation rates between non-directed and directed donors. The protocol was approved by our institutional review board (7053-20-SMC).

### Pre-Donation Evaluation

The evaluation process for donors involves a comprehensive medical, social, and psychological assessment. Directed donors are subject to approval by a local independent committee at the Sheba Medical Center, while non-directed donors are referred to a national independent committee. Before deciding on a particular donor-recipient pair, the relevant committee requests information from the transplant center about the potential recipient and the donor as well as an independent psychological evaluation. It is important to note that the transplant center medical team provides all donors with the assurance that they can choose to withdraw from the donation process at any point, without any guilt or negative consequences.

### Primary and Secondary Outcomes

Our primary outcomes were the physical component summary (PCS) score and the mental component summary (MCS) score calculated from the 12-item Short Form Survey (SF-12) questions, with health outcomes grouped into eight domains, namely, physical functioning, role-physical, bodily pain, general health, vitality, social functioning, role-emotional, and mental health [14]. These scores were normalized to a mean score of 50 and a standard deviation of 10 [15], meaning that a score of 50 represents the average HRQol of the general population, and a score of 40 or 60 represents a HRQol one standard deviation lower or higher, respectively, than the average. We conducted a comparative analysis of the PCS and MCS scores between non-directed and directed donors, and further investigated the variations in the eight domains that constitute the PCS and MCS scores for the two groups.

Donors participating in the study were required to provide written consent by answering “yes” to the first question on our questionnaire (Supplementary Figure S1). Before filling out the SF-12 questionnaire, participants were requested to respond to four additional questions regarding the time to return to normal activity post-donation, pre-donation exercising status, exercising status at the time of questionnaire completion, and time to return to exercising post-donation. In addition, we modified the SF-12 questionnaire by requesting participants to take “the day they reported being back to normal activity after kidney donation” as their baseline for answering questions, rather than “during the past 4 weeks,” as stated in the original questionnaire.

To fortify the HRQoL evaluation, we also compared several secondary outcomes between non-directed and directed donors. These outcomes are pertinent to HRQoL or influenced by it and include hospital LOS for kidney donation, times to return to normal activity and physical activity post-donation, post-donation cessation of exercising, starting physical activity post-donation, and continuation of physical activity post-donation.

### Data Extraction and Study Assessments

The following information was extracted from donor medical records: donor type, smoking status and relevant family history—specifically of diabetes, hypertension, ischemic heart disease, malignancy, and nephrolithiasis. Additionally, donor information was obtained from the electronic patient records in the MDClone data acquisition system of the Sheba Medical Center. This system facilitated retrieval of relevant clinical information for donors, including age, gender, weight, and body mass index (BMI) pre-donation, hospital LOS for kidney donation, average systolic blood pressure and diastolic blood pressure in the 6 months pre-donation and in the first month post-donation. The following biochemical parameters were also retrieved from MDClone: average serum creatinine in the 6 months pre-donation and in the first week and 6 months post-donation, average uric acid in the 6 months pre-donation and in the first week post-donation, and average total white blood cell (WBC) count, hemoglobin, platelet count, globulins, albumin, glucose, HbA1C, lipid profile, urine protein/creatinine and urine albumin/creatinine in the 6 months pre-donation.

In view of the fact that both directed and non-directed donors are acquainted with their recipients (non-directed donors meet their recipients for the first time post-donation, during admission for kidney donation), we also determined whether there were any significant differences between the recipients of non-directed donors and directed donors that could impact the HRQoL of donors following donation. The following information about the recipients was retrieved from electronic records: transplant number, underlying cause of end-stage renal disease (ESRD), renal replacement therapy pre-transplant (yes/no), duration of dialysis, past medial history of diabetes, hypertension, ischemic heart disease, congestive heart failure, peripheral vascular disease, and malignancy, smoking status, human leukocyte antigen (HLA) match between the donor and recipient, delayed graft function (yes/no), slow graft function (yes/no), perioperative complications, and peri-transplant biopsy proven acute cellular rejections (BPACR). Additional clinical and biochemical parameters for the recipients were retrieved from MDClone, including age, gender, average weight and BMI for the 1–12 months post-transplant, serum creatinine on postoperative day 5, and at 1, 3, and 6 months and 1 year post-transplant.

### Statistical Analysis

Donors’ and recipients’ demographic, clinical and biochemical covariates of interest were tabulated and compared between non-directed and directed donors. Categorical variables were compared using the Chi-squared test, or Fisher’s test if the expected count number was less than 5. For continuous variables, we first checked for normality using the Shapiro-Wilks test and for equality of variances (using Levene’s test). We then used a t-test for normally distributed variables, and non-parametric tests (Mann-Whitney) for non-normally distributed variables. Differences in PCS and MCS index and components were analyzed using an independent sample t-test.

PCS score, MCS score and LOS were selected as the major dependent variables for linear regression analyses. The variables entered into the model were chosen after checking for multicollinearity and association with donation type. Variables that were significant (*p* ≤ 0.05) and/or those with clinical importance were entered into multivariate models. Logistic regression was also conducted for predicting PCS and MSC scores after dividing the index into two categories based on a threshold value of 50, followed by calculating the odds ratio (OR) with 95% CI. The data was analyzed using SPSS version 28.

## Results

### Donor Cohort Characteristics

A total of 112 living kidney donors comprised our final cohort. Mean age was 43.0 ± 10.7 years; 66 (58.9%) were males; and mean BMI was 24.2 ± 2.5 kg/m^2^. Of the donors, 30 (26.8%), 25 (22.5%), 24 (21.4%), 28 (25.0%), and 125 (10.7%) had a family history of diabetes, hypertension, ischemic heart disease, malignancy and nephrolithiasis, respectively; 14 (12.5%) were current smokers and 7 (6.3%) were past smokers. All cohort characteristics including average vital signs in the 6 months pre-donation and in the 1 month post-donation are shown in Table 1.

**TABLE 1:** Demographic and clinical characteristics of donors, stratified by donor type.

Variable	Entire cohort (*n* = 112)	Nondirected donors (*n* = 82)	Directed donors (*n* = 30)	*p*-value
Donor characteristic				
Age (years)	43.0 ± 10.7	43.1 ± 10.2	42.8 ± 12.2	0.45
Male sex	66 (58.9%)	57 (69.5%)	9 (30.0%)	<0.001**
Weight (kg)	71.4 ± 10.4	72.2 ± 9.8	69.2 ± 11.9	0.09
BMI (kg/m^2^)	24.2 ± 2.5	24.1 ± 2.4	24.5 ± 2.7	0.25
Family history of diabetes	30 (26.8%)	18 (22.2%)	12 (40.0%)	0.056
Family history of hypertension	25 (22.5%)	16 (19.5%)	9 (31.0%)	0.202
Family history of ischemic heart disease	24 (21.4%)	14 (17.1%)	10 (33.3%)	0.063
Family history of malignancy	28 (25.0%)	20 (24.4%)	8 (26.7%)	0.805
Family history of nephrolithiasis	12 (10.7%)	7 (8.5%)	5 (16.7%)	0.218
Smoking status				
Current smoker	14 (12.5%)	8 (9.7%)	6 (20.0%)	0.338
Past smoker	7 (6.3%)	5 (6.1%)	2 (6.7%)	
Never smoked	91 (81.3%)	69 (84.1%)	22 (73.3%)	
Average vital signs in the 6 months pre-donation				
SBP	122.6 ± 10.3	123.4 ± 10.5	120.3 ± 9.6	0.087
DPB	75.7 ± 7.3	76.0 ± 6.9	74.6 ± 8.4	0.091
Average vital signs in the first month post-donation				
SBP (max)	145.8 ± 15.5	145.9 ± 16.1	145.7 ± 13.9	0.481
SBP (min)	79.3 ± 14.0	78.7 ± 13.4	80.9 ± 15.7	0.239
SBP (average)	108.7 ± 10.2	108.8 ± 9.9	108.5 ± 11.3	0.448
DBP (max)	87.0 ± 9.1	86.2 ± 7.9	89.1 ± 12.0	0.118
DBP (min)	40.6 ± 9.4	40.6 ± 8.6	40.5 ± 11.6	0.483
DBP (average)	62.1 ± 8.4	62.0 ± 8.2	62.6 ± 8.9	0.369

Abbreviations: BMI, body mass index; DBP, diastolic blood pressure; SBP, systolic blood pressure.

Continuous variables are presented as means ± standard deviations, categorical variables are presented as numbers (%).

*p < *0.05*; **p < *0.01*.

The bold values are all the *p* values which are significant, either below 0.05 or below 0.01.

Of the 112 donors, 90 (80.4%) were healthy without any past medical history. Relevant past medical histories of 22 donors (14 non-directed and 8 directed) included hypertension (in 2), prediabetes (in 2), dyslipidemia (in 5), hypothyroidism (in 4), bariatric surgery (in 2), asthma (in 2), osteoporosis (in 1), celiac disease (in 1), motor cerebral palsy (in 1) and full recovery from breast carcinoma (in 1 directed donor). None of the donors had any mental disorder. Laboratory results including renal function tests of all donors in the 6 months pre-donation and at 1 week and 6 months post-donation are shown in Table 2.

**TABLE 2:** Biochemical characteristics of donors, stratified by donor type.

Variable	Entire cohort (*n* = 112)	Non-directed donors (*n* = 82)	Directed donors (*n* = 30)	*p*-value
Average laboratory results in the 6 months pre-donation				
WBC (K/μL)	7.4 ± 4.9	6.5 ± 1.4	10.0 ± 8.9	0.023*
Hemoglobin (g/dL)	14.0 ± 1.1	14.2 ± 1.0	13.6 ± 1.3	0.017*
Platelets (K/μL)	216.5 ± 43.3	213.0 ± 40.0	226.3 ± 50.9	0.082
Creatinine (mg/dL)	0.83 ± 0.15	0.85 ± 0.15	0.75 ± 0.15	0.002**
eGFR (CKD-EPI)^a^	101.7 ± 13.5	100.9 ± 13.9	103.8 ± 12.1	0.159
CCT urine collection (mL/min)	130.1 ± 25.5	132.1 ± 23.6	122.2 ± 31.7	0.085
Glucose (mg/dL)	90.9 ± 7.9	90.9 ± 6.4	90.8 ± 11.2	0.486
HbA1C (g/dL)	5.1 ± 0.4	5.1 ± 0.4	5.0 ± 0.5	0.105
Albumin (g/dL)	4.4 ± 0.3	4.5 ± 0.3	4.4 ± 0.3	0.03*
Globulins (g/dL)	2.8 ± 0.3	2.7 ± 0.3	2.8 ± 0.3	0.063
Uric acid (mg/dL)	5.2 ± 1.3	5.4 ± 1.2	4.6 ± 1.1	0.025*
Total cholesterol (mg/dL)	174.5 ± 27.7	171.1 ± 27.6	184.6 ± 26.4	0.042*
LDL cholesterol (mg/dL)	109.4 ± 22.7	106.6 ± 22.9	118.2 ± 20.3	0.027*
HDL cholesterol (mg/dL)	53.1 ± 11.3	51.5 ± 8.9	58.1 ± 16.0	0.055
Triglycerides (mg/dL)	88.6 ± 43.2	86.4 ± 40.9	95.5 ± 50.3	0.208
Urine protein/creatinine (g/g creatinine)	0.06 ± 0.03	0.06 ± 0.03	0.08 ± 0.05	0.083
Urine albumin/creatinine (mg/g creatinine)	3.8 ± 4.4	3.2 ± 3.7	5.8 ± 6.1	0.03**
Laboratory results in the first week post-donation				
Uric acid (mg/dL) average	4.3 ± 1.1	4.5 ± 1.1	3.8 ± 1.0	0.002**
Creatinine (mg/dL) max	1.4 ± 0.3	1.41 ± 0.3	1.2 ± 0.2	0.001**
Creatinine (mg/dL) min	1.3 ± 0.3	1.3 ± 0.3	1.1 ± 0.2	<0.001**
Creatinine (mg/dL) average	1.3 ± 0.3	1.4 ± 0.3	1.2 ± 0.2	<0.001**
eGFR average (CKD-EPI)^a^	61.3 ± 11.1	59.5 ± 10.8	67.0 ± 10.4	0.002**
Laboratory results in the 6 months post-donation				
Creatinine (mg/dL)	1.2 ± 0.2	1.2 ± 0.2	1.1 ± 0.02	0.034*
eGFR (CKD-EPI)^a^	69.3 ± 14.3	69.3 ± 14.8	69.0 ± 12.3	0.464

Abbreviations: WBC, white blood cell; CCT, creatinine clearance; eGFR, estimated glomerular filtration rate.

Continuous variables are presented as means ± standard deviations, categorical variables are presented as numbers (%).

*p < 0.05; **p < 0.01.

^a^ eGFR was calculated according to the following CKD-EPI formula: eGFR = 141* min (Scr/k, 1)α * max (Scr/k, 1)−1.209 * 0.993Age * 1.018 * 1.159 (if black) (where Scr—standardized serum creatinine; k = 0.7 if female, 0.9 if male; α = −0.329 if female, −0.411 if male; min = the minimum of Scr/k of 1; max = the maximum of Scr/k or 1).

The bold values are all the *p* values which are significant, either below 0.05 or below 0.01.

### Univariate Comparison of Non-Directed vs. Directed Donors

Our cohort consisted of 82 non-directed donors and 30 directed donors. Directed donors comprised 22 (73.3%) living related donors (8 daughters, 6 sons, 6 sisters, and 2 brothers) and 8 (26.7%) living unrelated donors (6 wives, 1 nephew, and 1 friend) (Figure 1). There were significantly more males among non-directed vs. directed donors (69.5% vs. 40%, *p* < 0.001). Rates of family history of diabetes and of ischemic heart disease were higher among directed compared to non-directed donors (40% vs. 22.2% and 33.3% vs. 17.1%, respectively, with *p* values approaching significance). There were no other differences between non-directed and directed donors, including no statistically significant differences in systolic and diastolic blood pressures in the 6 months pre-donation and 1 month post-donation, as shown in Table 1.

Biochemical characteristics differed between non-directed and directed donors in pre-donation total WBC count (6.5 ± 1.4 vs. 10.0 ± 8.9, *p* = 0.023) and in lipid profile (total cholesterol and LDL cholesterol), which were both higher in directed donors. Urine albumin/creatinine was higher in directed compared to non-directed donors, but values were in the normal range for both groups. The variations observed in hemoglobin, uric acid, albumin, creatinine and eGFR between non-directed and directed donors were primarily due to gender differences, with the higher proportion of female donors among the directed group contributing to lower levels of hemoglobin, uric acid, albumin and creatinine. All other biochemical characteristics were not statistically different between the two groups, as shown in Table 2.

### Univariate Comparison for Renal Transplant Recipients Who Received a Kidney From Non-Directed vs. Directed Donors

Renal transplant recipients (RTRs) who received a kidney from a non-directed vs. a directed donor were younger (49.7 ± 13.4 years vs. 56.1 ± 13.2 years, *p* = 0.013), had spent a longer time on dialysis pre-transplant [1.8 years (0.8–3.5) vs. 0.7 (0.3–2.0) years, *p* = 0.002], and exhibited a lower rate of hypertension (75.6% vs. 93.3%, *p* = 0.037) and a higher degree of human leukocyte antigen mismatch (HLA MM) (5–6 MM in 55.6% vs. 25.9% and 0% 0–2 MM vs. 25.9%, *p* < 0.001). No statistically significant differences were observed in hospital LOS, rates of delayed or slow graft function, peri-operative complications and peri-transplant BPACR between the two groups. All other demographic and clinical characteristics are shown in Table 3. Renal allograft function on postoperative day 5, and at 1, 3, 6 and 12 months post-transplant did not differ significantly between the groups (Table 4).

**TABLE 3:** Demographic and clinical characteristics of renal transplant recipients, stratified by donor type.

Variable	Entire cohort (*n* = 112)	Non-directed donors (*n* = 82)	Directed donors (*n* = 30)	*p*-value
RTR characteristics				
Age (years)	51.4 ± 13.6	49.7 ± 13.4	56.1 ± 13.2	0.013*
Sex –Male	71 (63.4%)	52 (63.4%)	19 (63.3%)	0.994
Weight, average of 1–12 months post-transplant (kg)	75.8 ± 15.7	75.6 ± 16.2	76.4 ± 14.7	0.407
BMI, average of 1–12 months post-transplant (kg/m^2^)	26.8 ± 5.1	26.5 ± 5.0	27.4 ± 5.2	0.206
ESRD etiology				
Diabetic nephropathy	16 (14.3%)	11 (13.4%)	5 (16.7%)	0.13
Glomerulonephritis	25 (22.3%)	17 (20.7%)	8 (26.7%)	
Nephrosclerosis	10 (8.9%)	4 (4.9%)	6 (20.0%)	
PCKD	17 (15.2%)	14 (17.1%)	3 (10.0%)	
Other	27 (24.1%)	22 (26.8%)	5 (16.7%)	
Unknown	17 (15.2%)	14 (17.1%)	3 (10.0%)	
Pre-transplant dialysis				
Dialysis before transplant	84 (75.0%)	64 (78.0%)	20 (66.7%)	0.218
Time on dialysis (years)	1.4 (0.6–2.9)	1.8 (0.8–3.5)	0.7 (0.3–2.0)	0.002**
Medical history				
Diabetes	31 (27.7%)	20 (24.4%)	11 (36.7%)	0.198
Hypertension	90 (80.4%)	62 (75.6%)	28 (93.3%)	0.037*
Ischemic heart disease	21 (18.8%)	13 (15.9%)	8 (26.7%)	0.194
Congestive heart failure	12 (10.7%)	8 (9.8%)	4 (13.3%)	0.731
Peripheral vascular disease	4 (3.6%)	2 (2.4%)	2 (6.7%)	0.291
Malignancy	6 (5.4%)	4 (4.9%)	2 (6.7%)	0.658
Smoking status				
Current smoker	9 (8.1%)	4 (4.9%)	5 (16.7%)	0.124
Past smoker	23 (20.6%)	17 (20.7%)	6 (20.0%)	
Never smoked	80 (71.4%)	61 (74.4%)	19 (63.3%)	
Transplant number				
1	101 (90.2%)	74 (90.2%)	27 (90.0%)	0.496
2	7 (6.3%)	4 (4.9%)	3 (10.0%)	
3	3 (2.7%)	3 (3.7%)	0	
4	1 (1.2%)	0	0	
HLA MM				
0–2	7 (6.5%)	0	7 (25.9%)	<0.001**
3–4	49 (45.4%)	36 (44.4%)	13 (48.1%)	
5–6	52 (48.1%)	45 (55.6%)	7 (25.9%)	
Peri-transplant data				
Hospital LOS for transplant (days)	8 (8–10)	8 (8–10)	8.5 (8–10)	0.276
Delayed graft function	1 (0.9%)	0	1 (3.3%)	0.268
Slow graft function	10 (8.9%)	7 (8.5%)	3 (10.0%)	0.726
Peri-operation complications				
CVS	4 (3.6%)	4 (4.9%)	0	0.221
ID	18 (16.1%)	15 (18.3%)	3 (10.0%)	
Vascular	5 (4.5%)	2 (2.4%)	3 (10.0%)	
Other	1 (0.9%)	1 (1.2%)	0	
None	84 (75.0%)	60 (73.2%)	24 (80.0%)	
Peri-transplant BPACR	11 (9.8%)	10 (12.2%)	1 (3.3%)	0.283

Abbreviations: BMI, body mass index; BPACR, biopsy proven acute cellular rejections; CVS, cardiovascular; ESRD, end stage renal disease; HLA MM, human leukocyte antigen mismatch; ID, infectious diseases; PCKD, polycystic kidney disease; RTRs, renal transplant recipients.

Continuous variables are presented as means ± standard deviations, categorical variables are presented as numbers (%).

*p < 0.05; **p < 0.01.

The bold values are all the *p* values which are significant, either below 0.05 or below 0.01.

**TABLE 4:** Renal allograft function of renal transplant recipients, stratified by donor type.

Variable	Entire cohort (*n* = 112)	Non-directed donors (*n* = 82)	Directed donors (*n* = 30)	*p*-value
Postoperative day 5				
Creatinine (mg/dL) on	1.7 ± 1.3	1.7 ± 1.2	1.6 ± 1.6	0.379
1 month post-transplant				
Creatinine (mg/dL)	1.3 ± 0.4	1.3 ± 0.4	1.3 ± 0.4	0.452
eGFR (CKD-EPI)^a^	62.8 ± 21.3	63.2 ± 20.8	61.6 ± 22.4	0.366
3 months post-transplant				
Creatinine (mg/dL)	1.3 ± 0.7	1.4 ± 0.8	1.3 ± 0.4	0.283
eGFR (CKD-EPI)	63.7 ± 21.7	64.0 ± 21.2	63.0 ± 23.4	0.418
6 months post-transplant				
Creatinine (mg/dL)	1.3 ± 0.6	1.3 ± 0.7	1.2 ± 0.4	0.267
eGFR (CKD-EPI)	65.7 ± 20.1	65.8 ± 19.5	65.6 ± 21.9	0.474
1 year post-transplant				
Creatinine (mg/dL)	1.3 ± 0.8	1.3 ± 0.9	1.2 ± 0.4	0.231
eGFR (CKD-EPI)	68.3 ± 20.0	67.8 ± 19.8	69.6 ± 21.0	0.254

Abbreviations: eGFR, estimated glomerular filtration rate.

Continuous variables are presented as means ± standard deviations.

^a^ eGFR was calculated according to the following CKD-EPI formula: eGFR = 141* min (Scr/k, 1)α * max (Scr/k, 1)−1.209 * 0.993Age * 1.018 * 1.159 (if black) (where Scr—standardized serum creatinine; k = 0.7 if female, 0.9 if male; α = −0.329 if female, −0.411 if male; min = the minimum of Scr/k of 1; max = the maximum of Scr/k or 1).

### Primary and Secondary Outcomes in the Entire Donor Cohort

Mean time from donation to questionnaire completion was 1.07 ± 0.65 years. Mean PCS-12 and MCS-12 scores were both higher than those in the general population (54.1 ± 4.1 and 55.5 ± 5.8, respectively). Median time to normal activity post-donation was 30 days [interquartile range (IQR) 14–42]. Of the donors, 77 (68.8%) reported exercising before donation and 78 (69.6%) post-donation, with a median time to physical activity post-donation of 48 days (IQR 30–90); 66 (58.9%) continued exercising, 11 (9.8%) stopped exercising, and 12 (10.7%) started physical activity post-donation. Mean hospital LOS for kidney donation was 3.7 ± 0.9 days (Table 5).

**TABLE 5:** Primary and secondary outcomes for donors, stratified by donor type.

Variable	Entire cohort (*n* = 112)	Non-directed donors (*n* = 82)	Directed donors (*n* = 30)	*p*-value
Primary outcome—questionnaire results				
Time from donation to questionnaire completion (years)	1.07 ± 0.65	1.02 ± 0.56	1.21 ± 0.85	0.141
PCS-12 score	54.1 ± 4.1	55.1 ± 3.1	51.1 ± 5.2	<0.001**
MCS-12 score	55.5 ± 5.8	56.9 ± 4.1	51.8 ± 7.9	<0.001**
Secondary outcomes				
Time to normal activity post-donation (days)	30 (14–42)	30 (14–40)	30 (16–45)	0.117
Physical activity before donation	77 (68.8%)	58 (70.7%)	19 (63.3%)	0.454
Physical activity after donation	78 (69.6%)	61 (74.4%)	17 (56.7%)	0.071
Time to physical activity post-donation (days)	48 (30–90)	45 (30–90)	60 (34–90)	0.306
Change in physical activity post-donation				
Continued	66 (58.9%)	52 (63.4%)	14 (46.7%)	0.287
Stopped	11 (9.8%)	6 (7.3%)	5 (16.7%)	
Started	12 (10.7%)	9 (11.0%)	3 (10.0%)	
Never did	23 (20.5%)	15 (18.3%)	8 (26.7%)	
Hospital LOS for kidney donation (days)	3.7 ± 0.9	3.4 ± 0.7	4.4 ± 1.1	<0.001**

Continuous variables are presented as means ± standard deviations or as median (interquartile range), categorical variables are presented as numbers (%).

**p < 0.01.

The bold values are all the *p* values which are significant, either below 0.05 or below 0.01.

### Univariate Comparison of Primary and Secondary Outcomes in Non-Directed vs. Directed Donors

**FIGURE 2:**
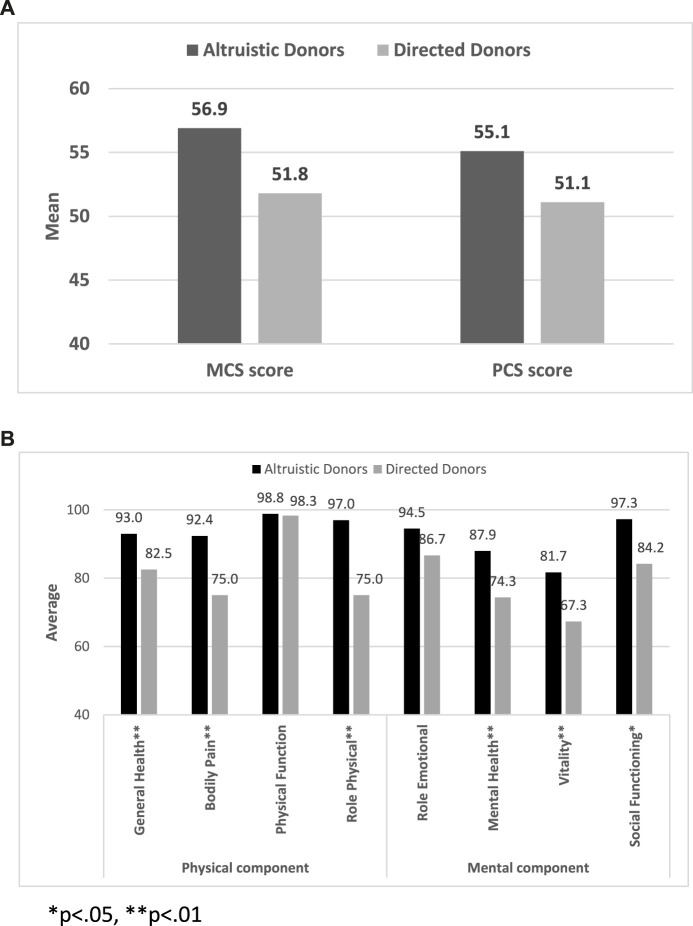
SF-12 questionnaire results: **(A)** Mean MCS-12 and PCS-12 scores for non-directed vs. directed donors. **(B)** PCS-12 and MCS-12 scores for non-directed vs. directed donors for the eight domains of the SF-12 questionnaire.

**FIGURE 3:**
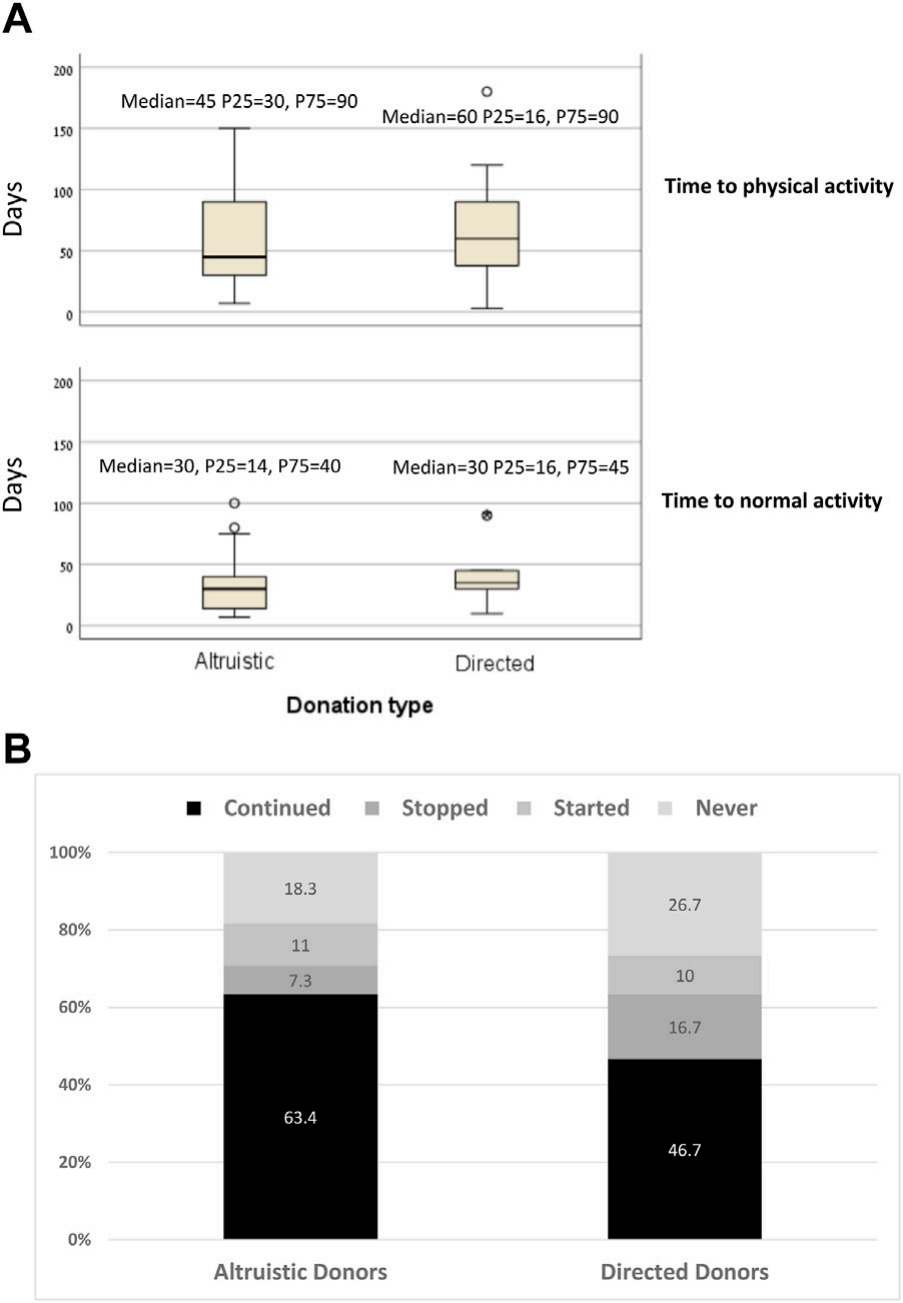
**(A)** Time to normal activity and to physical activity for non-directed vs. directed donors. **(B)** Rates of kidney donors who continued, stopped, started exercising after donation and of donors who did not exercise before or after donation for non-directed vs. directed donors.

**FIGURE 4:**
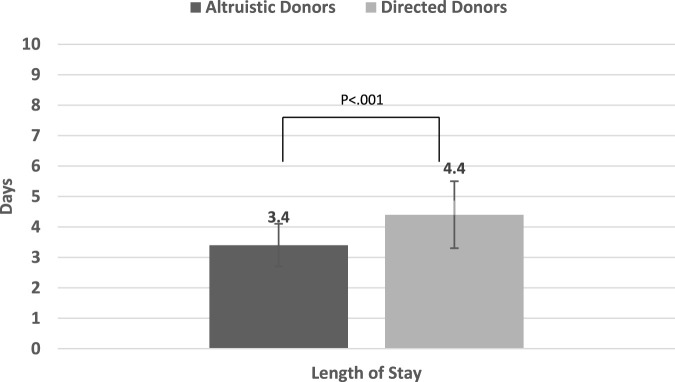
Mean in-hospital length of stay (LOS) for kidney donation for non-directed vs. directed donors.

Comparisons for all primary and outcomes secondary are presented in Table 5. Mean PCS-12 and MCS-12 scores were significantly higher in non-directed compared to directed donors (55.1 ± 3.1 vs. 51.1 ± 5.2, *p* < 0.001 and 56.9 ± 4.1 vs. 51.8 ± 7.9, *p* < 0.001, respectively) (Figure 2A). There were also significant differences between the two groups in six of the eight domains of the SF-12 questionnaire (general health, bodily pain, and role physical for the PCS score, and mental health, vitality, and social functioning for the MCS score) (Figure 2B). Time to resumption of normal activity was not significantly different between the two groups. However, time to resumption of physical activity was shorter for the non-directed donors than for the directed donors [45 days (IQR 30–90) vs. 60 days (IQR 34–90)], but significant difference could not be shown due to the small size of the two groups (Figure 3A). More non-directed than directed donors engaged in physical activity before and after kidney donation and continued exercising post-donation. A higher rate of directed vs. non-directed donors did not exercise before or after kidney donation or stopped exercising post-donation (Figure 3B). Hospital LOS for kidney donation was significantly longer for directed than for non-directed donors (4.4 ± 1.1 vs. 3.4 ± 0.7 days, *p* < 0.001) (Figure 4).

### Multivariable Linear Regression Analysis of PCS-12 Score in Kidney Donors

In a multivariable linear regression analysis of the PCS-12 score in kidney donors (adjusted for donor type, age, gender, donor family history of diabetes and of ischemic heart disease, average eGFR in the first week post-donation, WBC count in the 6 months pre-donation, and hospital LOS for kidney donation), only donor type and WBC count were found to be significant predictors for PCS-12 score. Being a non-directed donor vs. a directed donor is associated with a 2.69 (1.02) points higher mean PCS-12 score, *p* = 0.01. For every increase of 1 K/μL in WBC count in the 6 months pre-donation, PCS-12 score decreased by 0.18 (0.08), (*p* = 0.02; Table 6).

**TABLE 6:** Multivariate linear regression analysis for PCS-12, MCS-12 and hospital LOS for kidney donors.

Effect	Mean (SD)	*p*-value
Multivariate linear regression analysis for PCS-12		
Donor type (non-directed vs. directed)	2.69 (1.02)	0.01*
Age (for every increase of 1 year)	0.02 (0.04)	0.58
Gender (male vs. female)	(−)0.33 (0.90)	0.72
Donor family history of diabetes (Yes vs. No)	(−)0.07 (0.90)	0.94
Donor family history of ischemic heart disease (Yes vs. No)	(−)0.82 (0.95)	0.39
eGFR average in the first week post-donation (for every increase of 1 mL/min)	0.02 (0.04)	0.6
WBC count in the 6 months pre-donation (for every increase of 1 K/μL)	(−)0.18 (0.08)	0.02*
Hospital LOS for kidney donation (for every increase of 1 day)	(−)0.14 (0.46)	0.77
Multivariate linear regression analysis for MCS-12		
Donor type (non-directed vs. directed)	4.43 (1.53)	0.005**
Age (for every increase of 1 year)	0.01 (0.07)	0.89
Gender (male vs. female)	(−)0.19 (1.35)	0.89
Donor family history of diabetes (Yes vs. No)	(−)0.20 (1.35)	0.88
Donor family history of ischemic heart disease (Yes vs. No)	0.58 (1.43)	0.69
eGFR average in the first week post-donation (for every increase of 1 mL/min)	0.07 (0.06)	0.24
WBC count in the 6 months pre-donation (for every increase of 1 K/μL)	(−)0.23 (0.12)	0.05
Hospital LOS for kidney donation (for every increase of 1 day)	(−)1.22 (0.69)	0.08
Multivariate linear regression analysis for hospital LOS for kidney donation		
Donor type (non-directed vs. directed)	(−)0.78 (0.22)	<0.001**
Age (for every increase of 1year)	(−)0.01 (0.01)	0.13
Gender (male vs. female)	(−)0.20 (0.20)	0.33
Donor family history of diabetes (Yes vs. No)	0.54 (0.19)	0.007**
Donor family history of ischemic heart disease (Yes vs. No)	0.00 (0.22)	1.00
eGFR average in the first week post-donation (for every increase of 1 mL/min)	(−)0.01 (0.01)	0.44
WBC count in the 6 months pre-donation (for every increase of 1 K/μL)	0.01 (0.02)	0.67

Abbreviations: eGFR, estimated glomerular filtration rate; WBC, white blood cell.

*p < 0.05; **p < 0.01.

The bold values are all the *p* values which are significant, either below 0.05 or below 0.01.

### Multivariable Linear Regression Analysis of MCS-12 Score in Kidney Donors

In a multivariable linear regression analysis of the MCS-12 score in kidney donors adjusted for the same variables as those listed above, donor type alone was found to be a significant predictor for MCS-12 score. Mean MCS-12 score increased by 4.43 (1.53) in non-directed compared to directed donors (*p* = 0.005). Increases in WBC counts pre-donation and in hospital LOS for kidney donation reduced the MCS-12 score, with *p* values approaching significance (Table 6).

### Multivariable Linear Regression Analysis of Hospital LOS for Kidney Donation

In a multivariable linear regression analysis of hospital LOS for kidney donation adjusted for the same variables as those listed above, donor type and family history of diabetes were found to be significant predictors for LOS. LOS was shorter by 0.78 (0.22) days in non-directed compared to directed donors (*p* < 0.001). Family history of diabetes prolonged the LOS by 0.54 (0.19) days (*p* = 0.007; Table 6). There were no intraoperative surgical problems or any postoperative complications during hospitalization in our study cohort of living kidney donors.

### Multivariable Logistic Regression Analysis of PCS-12 and MCS-12 Scores Above 50 in Kidney Donors

In a multivariable logistic regression analysis of PCS-12 and MCS-12 scores above 50 adjusted for the same variables as those listed above, donor type alone was found to be significantly associated with PCS-12 and MCS-12 score above 50. The odds for PCS-12 score to be above 50 were almost 10 times higher in non-directed compared to directed donors (OR 9.9, 95% CI 1.48–66, *p* = 0.018). The odds for an MCS-12 score above 50 were more than 16 times higher in non-directed vs. directed donors (OR 16.23, 95% CI 2.37–111.02, *p* = 0.005).

## Discussion

As the number of live kidney donations, particularly non-directed donations, continues to rise, it is becoming imperative to conduct a comprehensive analysis of donor outcomes, including a thorough comparison of outcomes between non-directed and directed donors in terms of both physical and mental health, as reflected in HRQol.

Our assessment of HRQol was based on a variety of factors, primarily PCS-12 and MCS-12 scores, but also time to resumption of normal activity, changes in the rate of physical activity, and the time taken to return to physical activity after donation. Our findings indicate that live kidney donors experience better HRQol than the general population with mean PCS-12 and MCS-12 scores surpassing the average score of 50. The median time for donors to return to normal activity and to physical activity was 30 and 48 days, respectively, and 58.9% of donors continued to exercise post-donation, while another 10.7% started exercising post-donation. Our analysis revealed that non-directed donors had a significantly higher HRQol than directed donors, as demonstrated by both PCS-12 and MCS-12 scores. Moreover, a higher proportion of non-directed donors continued with physical activity and they resumed exercising sooner after donation compared to directed donors. Mean hospital LOS for kidney donation was 3.7 days, with LOS being significantly shorter for non-directed than for directed donors. Our multivariable analyses demonstrated that non-directed donation was an independent predictor of higher PCS-12 and MCS-12 scores as well as a shorter hospital LOS.

The literature shows that, in general, most living donors exhibit excellent medical heath and enjoy high levels of HRQol [16–21]. However, studies investigating the psychological outcomes after non-directed kidney donation are limited. Sadler et al. conducted an early investigation (1971) of 18 living unrelated kidney donors that revealed that the donors did not exhibit any unusual characteristics or significant mental illness during the donation process. However, a retrospective follow-up conducted 4–6 years later showed that three of the donors had developed psychiatric disorders, including two cases of alcoholism and one of anti-social personality disorder [22]. A later study of 24 non-directed donors reported a considerable positive impact of donation on psychological wellbeing and very high satisfaction with the donation [23]. However, in another study of 49 unspecified living donors, psychologic symptoms increased after donation [24]. In the only study to date comparing non-directed donors to directed donors (39 vs. 52), similar positive outcomes were observed after donation. The majority of non-directed donors reported feeling content with the donation process and expressed a strong willingness to make the same decision again, with the caveat that three non-directed donors did regret their decision to donate [25]. Our study is the first to demonstrate superior HRQol experienced by a substantial group of non-directed donors compared to directed donors.

In our study, the significant disparity in the MCS-12 score between non-directed and directed donors probably derives from the distinctive characteristics of the non-directed donor population in Israel. In Israel, most non-directed donors are Orthodox Jews whose “point of contact” is the Matnat Chaim organization. Their religious conviction to assist others and fulfill a righteous duty probably plays a crucial role in promoting non-directed donation, as saving person’s life is considered a significant religious obligation. This world view is exemplified by a passage in the Babylonian Talmud, Tractate Sanhedrin on page 37a, which states, “He who saves one life is as if he has saved the entire world.” Indeed, non-directed donors scored significantly higher in the mental health and vitality domains of the MCS-12 score (Figure 2B), suggesting that belief and faith contribute to feelings of calmness, completeness, and energy. Furthermore, non-directed donors exhibited better social functioning than directed donors. While it is possible that the strong religious faith of non-directed donors makes them mentally more resilient than directed donors, further research is required to confirm this premise.

Non-directed donors showed higher energy levels and better PCS-12 scores, potentially explaining the shorter time to the resumption of physical activity post-donation, the greater likelihood of continuing physical activity and initiating exercise after donation compared to directed donors. In terms of the duration of hospital stay post-donation, patients’ complaints of pain and willingness to extend their stay were the main factors determining LOS in the absence of any surgical or post-operative complications. Notably, non-directed donors had a shorter hospital stay, probably due to their faster physical recovery associated with less pain (Figure 2B) and their superior mental wellbeing.

Interestingly, an increase in WBC count was found to be associated with the PCS-12 score. This finding is in line with prior research demonstrating a link between excessive inflammatory activity and physical health problems, including cardiovascular disease, stroke, certain cancers and autoimmune disorders [26], with substantial morbidity and mortality being attributable to inflammation-related conditions [27, 28]. Donor family history of diabetes was found to be associated with an increase in hospital LOS. This observation has no obvious explanation currently.

When interpreting our findings, it is important to consider the study’s strengths and its limitations. The strengths include the use of the widely validated SF-12 questionnaire, which provides a strong foundation for evaluating HRQol. The study sample is comprised of a large group of donors, which enhances the reliability of the findings. Additionally, by examining both donor and recipient characteristics, this study was able to consider multiple confounders, including clinical and biochemical factors collected both before and after donation or transplantation. However, additional confounders cannot be excluded. An additional limitation is that the use of patient questionnaires can introduce subjective elements, which can be a drawback compared to direct assessments of inpatients. Living donors are a select group chosen for their good health and we did not evaluate the HRQol of the donors prior to donation; it is thus possible that these donors already had good HRQol before donation and any improvement was not necessarily linked to the kidney donation itself. It is also possible that those who declined to participate or those who we could not reach would have affected our psychosocial and functional outcomes had they been included in the study.

Importantly, the findings of this study endorse the continued use of non-directed donors, given the enhanced physical and mental HRQoL observed after donation, indicating that the donation process has no negative impact on their physical or mental wellbeing. In fact, carefully screened donors do not suffer any adverse physical or psychological consequences from donating to a stranger. Nevertheless, it is crucial to emphasize the benefits of living related donors, such as the improved HLA matching within families that leads to lower rejection rates and improved long-term outcomes. As healthcare providers, we strongly believe that safeguarding the wellbeing of all donors, particularly those motivated by altruism, is our fundamental duty. To minimize the risk of adverse health consequences post-donation and prevent any potential future harm, selecting non-directed donors should involve meticulous screening and a more stringent process. Moreover, it is imperative to ensure that the eagerness of non-directed donors to help others is not exploited or manipulated in any way. Therefore, the use of non-directed kidney donation should be considered only as a last resort after exhausting all possible options to secure a donation within the family.

## Data Availability

The raw data supporting the conclusion of this article will be made available by the authors, without undue reservation.

## Abbreviations

BMI, body mass index; BPACR, biopsy proven acute cellular rejections; eGFR, estimated glomerular filtration rate; ESRD, end stage renal disease; HLA MM, human leukocyte antigen mismatch; IQR, interquartile range; LOS, length of stay; MCS, mental component summary; OR, odds ratio; PCKD, polycystic kidney disease; PCS, physical component summary; RTRs, renal transplant recipients; Scr, serum creatinine; SF-12, 12-item Short Form Survey; WBC, white blood cell.
