# Supplementary material for: Assessing Health-Related Quality of Life in Non-Directed Versus Directed Kidney Donors: Implications for the Promotion of Non-Directed Donation
Source: Transpl Int. 2024 Jan 12;37:12417. doi: 10.3389/ti.2024.12417 (PMC10811092; doi:10.3389/ti.2024.12417)
Supplement: Supplementary file 1 [file DataSheet1.docx]

SUPPLEMENTARY FILE

FIGURE 1 Modified SF-12 questionnaire with 4 supplementary questions

**Patient name: ID: Date:**

**Do you agree the questionnaire will be used for research purposes?** YES NO

**1. How long did it take you to return to normal activity after kidney donation?___** days

**2. Did you exercise before kidney donation?** YES NO

**3. Do you exercise at present?** YES NO

**4. If you exercise at present, how long did it take you to return to exercising after kidney donation? ____** days

**SF-12 Health Survey** This survey asks for your views about your health. This information will help keep track of how you feel and how well you are able to do your usual activities. **Answer each question by choosing just one answer**. If you are unsure how to answer a question, please give the best answer you can.

**Please answer the following questions starting from the day you reported you were back to normal after kidney donation** ____________________________________________________________________

1. **In general, would you say your health is**:

**□**1 Excellent **□**2 Very good **□**3 Good **□**4 Fair **□**5 Poor

**The following questions are about activities you might do during a typical day.**

**From when you got back to normal after kidney donation, does your health limit you in these activities? If so, how much?**

**□**1 YES, limited a lot **□**2 YES, limited a little **□**3 NO, not limited at all

2. Moderate activities such as moving a table, pushing a vacuum cleaner, bowling, or playing golf. □1 □2 □3

3. Climbing several flights of stairs. **□**1 **□**2 **□**3

**From when you got back to normal after kidney donation, have you had any of the following problems with your work or other regular daily activities as a result of your physical health?**

**□**1 YES **□**2 NO

4. Accomplished less than you would like. □1 □2

5. Were limited in the kind of work or other activities. □1 **□**2

**From when you got back to normal after kidney donation, have you had any of the following problems with your work or other regular daily activities as a result of any emotional problems (such as feeling depressed or anxious)?**

**□**1 YES **□**2 NO

6. Accomplished less than you would like. □1 □2

7. Did work or activities less carefully than usual. □1 □2

8. **From when you got back to normal after kidney donation, how much did pain interfere with your normal work (including work outside the home and housework)?**

**□**1 Not at all **□**2 A little bit **□**3 Moderately **□**4 Quite a bit **□**5 Extremely

**These questions are about how you have been feeling** **from when you got back to normal after kidney donation. For each question, please give the one answer that comes closest to the way you have been feeling.**

**How much of the time from when you got back to normal after kidney donation …**

**□**1 All of the time **□**2 Most of the time **□**3 A good bit of the time **□**4 Some of the time **□**5 A little of the time **□**6 None of the time

9. Have you felt calm & peaceful? **□**1 **□**2 **□**3 **□**4 **□**5 **□**6

10. Did you have a lot of energy? **□**1 **□**2 □3 **□**4 **□**5 **□**6

11. Have you felt down-hearted and blue? **□**1 **□**2 □3 **□**4 **□**5 **□**6

12. **From when you got back to normal after kidney donation, how much of the time has your physical health or emotional problems interfered with your social activities (like visiting friends, relatives, etc.)?**

**□**1 All of the time **□**2 Most of the time **□**3 Some of the time **□**4 A little of the time **□**5 None of the time
